# Effect of Repeated Anodal HD-tDCS on Executive Functions: Evidence From a Pilot and Single-Blinded fNIRS Study

**DOI:** 10.3389/fnhum.2020.583730

**Published:** 2021-01-18

**Authors:** Hongliang Lu, Yue Gong, Peng Huang, Yajuan Zhang, Zhihua Guo, Xia Zhu, Xuqun You

**Affiliations:** ^1^Faculty of Medical Psychology, Air Force Medical University, Xi’an, China; ^2^School of Psychology, Shaanxi Normal University, Xi’an, China

**Keywords:** executive functions, tDCS, fNIRS, inhibitory control, working memory, cognitive flexibility

## Abstract

Executive functions are of vital importance in the process of active cognition, which is thought to be associated with the dorsolateral prefrontal cortex (DLPFC). As a valid brain stimulation technology, high-definition transcranial direct current stimulation (HD-tDCS) has been used to optimize cognitive function in healthy adults. Substantial evidence indicates that short-term or single anodal tDCS sessions over the left DLPFC will enhance the performance of executive functions. However, the changes in performance and cortical activation of executive functions after modulation by repeated anodal HD-tDCS is as yet unexplored. This study aims to examine changes in three core components of executive functions (inhibitory control, working memory, and cognitive flexibility) produced by nine HD-tDCS sessions (1.5 mA, over left DLPFC, 20 min per session), and to use functional near-infrared spectroscopy (fNIRS) to bilaterally record DLPFC neural activity. A total of 43 participants were divided randomly into two study groups (anodal group vs. sham group) to complete nine interventions. Our results demonstrate that the enhancement of cognitive flexibility in the anodal group was significantly better than that in the sham group. Additionally, a Stroop effect-related decrease in oxygenated hemoglobin (HbO) concentration in the DLPFC was observed in the anodal group but not the sham group. In conclusion, our study found that repeated anodal HD-tDCS sessions can significantly promote cognitive flexibility, one of the core components of executive function, and that alterations in DLPFC activation can enhance our understanding of the neuroplastic modifications modulated by HD-tDCS.

## Introduction

Executive functions play a key role in a series of top-down mental processes and coordinate various cognitive functions to complete prioritized tasks (Funahashi, 2001; Diamond, 2012). A defective executive will invariably cause impediments in cognitive function. Abnormal executive changes are considered to be relevant to the symptoms of numerous neuropsychiatric disorders (Konrad et al., 2006; Snyder, 2013; de Vries et al., 2014; Degutis et al., 2015). Furthermore, the successful performance of job or study tasks seemingly relies on well-developed executive control (Borella et al., 2010; Diamond, 2016). However, executive function is not steady-state and can be enhanced by various interventions. Diamond (2012) suggested that executive function was more likely to be improved by repeated sessions of cognitive training. The neural mechanism for this enhancement of executive function is not clear, but substantial evidence shows that the prefrontal cortex (PFC) is linked to inhibitory control (Bari and Robbins, 2013). The PFC is a significant mediator of the allocation of cognitive resources, and a specific area within the PFC, the dorsolateral PFC (DLPFC), is thought to be responsible for the modulation of executive control (Mansouri et al., 2009; Lara and Wallis, 2014). Evidence from functional magnetic resonance imaging (fMRI) indicates that DLPFC activation is essential for the implementation of cognitive control during the Stroop test (MacDonald et al., 2000). Previous studies have revealed that deficits in the DLPFC were the neurological basis of behavioral disinhibition. For example, the DLPFC showed a different pattern of activation in people with Alzheimer’s disease compared to a control group, and this may be associated with a deficit in attention control (Rosano et al., 2005). The control of cocaine-seeking behavior is also thought to be related to the PFC (Mihindou et al., 2013). Thus, we assume that excitability changes in the PFC (DLPFC) will influence executive control and that using an effective neurological intervention technology to modulate the activity of the cortex concerning specific behaviors is a meaningful way to understand executive control.

Transcranial direct current stimulation (tDCS) is a safe, painless, non-invasive, and effective brain intervention that is widely used in clinical therapeutics and is also documented to be capable of producing improvements in cognitive function in healthy individuals (Elsner et al., 2013; Segrave et al., 2015; Shin et al., 2015; Cachoeira et al., 2016). Anodal tDCS has a positive effect on increasing cortical excitability whereas cathodal tDCS produces the opposite effect (Nitsche and Paulus, 2000). According to the results of recent studies, anodal tDCS over the DLPFC was observed to produce enhancements in executive functions including working memory (Nikolin et al., 2015; Siniatchkin, 2017; Schwippel et al., 2018), inhibitory control (Soltaninejad et al., 2015; Angius et al., 2019), and cognitive flexibility (Nejati et al., 2020). Most studies to date have focused on the short-term effects of a single tDCS session. However, repeated tDCS sessions might provide more application value (Ljubisavljevic et al., 2016). One study has suggested that multiple anodal tDCS (10 sessions) can improve cognitive control in drug addicts (Alizadehgoradel et al., 2020). This benefit was also observed in another study that found improved executive control in individuals with a borderline personality disorder as a result of 10 active tDCS sessions (Molavi et al., 2020). However, single-session tDCS does not necessarily produce reliable benefits on cognition in healthy individuals (Horvath et al., 2015), but it has been suggested that multiple modulations of anodal tDCS might induce a cumulative effect on the improvement of cognitive function (Christova et al., 2015). Also, the current flow of conventional tDCS was observed to spread to peripheral brain regions outside the targeted cortex (Keeser et al., 2011; Stagg et al., 2013), and the specific effect of a particular intervention is thus complicated and difficult to explain. High-definition transcranial direct current stimulation (HD-tDCS) is more efficient than conventional tDCS at producing current input with high density and high spatial precision on the cerebral cortex (Kuo et al., 2013). Moreover, anodal HD-tDCS over the left DLPFC has been proven to significantly enhance executive functions during conflict-related tasks, whereas stimulation of the right DLPFC or sham stimulation does not (Dubreuil-Vall et al., 2019). To obtain robust and precise enhancement of executive control in healthy participants, the strategy of repeated active HD-tDCS over the left DLPFC is worth exploring further.

Neuronal activity is related to local blood flow, and this vital mechanism of neurovascular coupling underlies functional imaging techniques that are used to measure neural activity (David and Costantino, 2002; O’Herron et al., 2016). As a noninvasive optical imaging technique, functional near-infrared spectroscopy (fNIRS) can quantify the concentration of oxyhemoglobin (HbO) and deoxyhemoglobin (HbR) by variations in light intensity, which are obtained by emitting continuous-wave light (650–950 nm) through the skull into the brain (Li et al., 2011; Boas et al., 2014; Pinti et al., 2020). Compared to conventional techniques, such as fMRI and positron emission tomography, fNIRS has relatively greater tolerance to movement artifacts and a high temporal sampling rate and is a more economical and portable way to continuously detect hemodynamic variations. In recent years, fNIRS has been applied to examine neural activation both in patients suffering from psychiatric or cognitive disorders and in healthy individuals (Abdalmalak et al., 2017; Nishizawa et al., 2019; Pinti et al., 2020). There is sufficient evidence to indicate that fNIRS is a promising technique for monitoring hemodynamic variation in the targeted cortical region during and after tDCS intervention (McKendrick et al., 2015; Choe et al., 2016; Muthalib et al., 2016).

This study aimed to examine behavioral changes in executive functions produced across repeated HD-tDCS sessions (nine sessions over 3 weeks) between anodal and control tDCS groups and to detect activation of focal cortical regions by fNIRS. Diamond (2012) suggested that executive control should consist of three core components: inhibitory control, working memory, and cognitive flexibility. Inhibitory control of attention (selective attention) plays a special role in bridging the other two components and is considered to be an indispensable basis for the process of cognitive flexibility (Davidson et al., 2006; Diamond, 2012) and to have an overlapping neural mechanism with working memory (Awh and Jonides, 2001; Gazzaley and Nobre, 2012). Therefore, it was crucial to investigate the variations in neural activity related to the selective attention function after HD-tDCS. Also, hemodynamic variations in the left DLPFC (stimulated cortex) and right DLPFC (unstimulated cortex) were recorded and analyzed in the present study, because the unilateral HD-tDCS intervention has been proven to enhance interhemispheric connectivity (Yaqub et al., 2018). We hypothesized that repeated active high-definition transcranial direct current stimulation (nine HD-tDCS sessions over 3 weeks) on the left DLPFC would improve executive functions and bilaterally change hemodynamic variation in both DLPFC during a selective attention task.

## Materials and Methods

### Participants

A total of 43 right-handed healthy undergraduates (mean age = 20.91 years, SD = 1.95 years; 24 males and 19 females) completed this study. They were randomly assigned to the anodal group and the sham group, and no significant differences were found in age after an independent-samples *t*-test, or in the educational background after a χ^2^-test (Table 1). Vision and hearing were normal or corrected in all participants. None had a history of neurological or psychiatric disorders or head injuries, and no participants were taking neuroleptic, hypnotic, or antiseizure medications that could influence neural activity. This study was approved by the Ethics Committee of Tangdu Hospital (2014-03-03) and has been registered with ClinicalTrials.gov (NCT02420470, http://www.clinicaltrials.gov/). All procedures were conducted according to the Declaration of Helsinki. All participants gave informed consent before the experiment and were paid after the experiment.

**Table 1:** Average ± standard deviation of demographic characteristics of participants.

	Anodal group (*n* = 22)	Sham group (*n* = 21)	*t* (or *χ*^2^)	*p*-value
Age (years)	20.73 ± 1.88	21.10 ± 2.05	−0.61	0.54
Education (years)	15.82 ± 1.94	16.14 ± 2.13	−5.23	0.60
Number (male/female)	12/10	12/9	0.02	0.88

### Cognitive Test

#### Color-Word Stroop Test (CW-Stroop Test)

**Figure 1:**
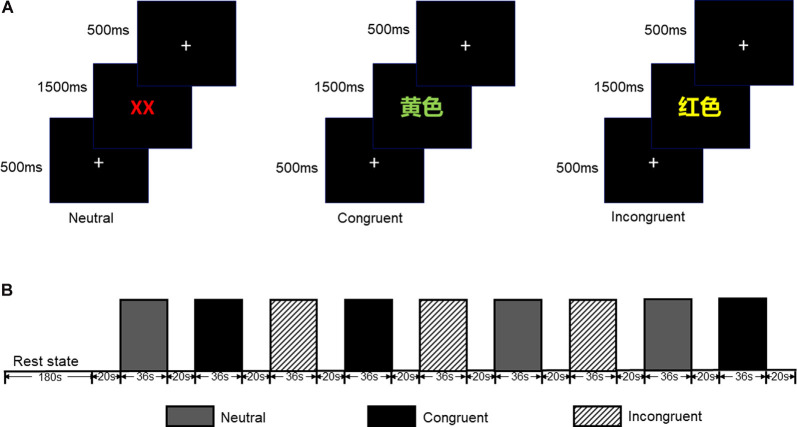
**(A)** Participants were required to identify the font color quickly and to ensure the accuracy of each reaction. **(B)** Each test block lasted 36 s while each rest block lasted 20 s (included 2 s cue); all test blocks were displayed in random order.

The CW-Stroop test is a widely used psychological test to measure inhibitory control (Vakil et al., 1995; Zhai et al., 2019). The task in our experiment was a block design with three conditions: incongruent, congruent, and neutral. The test stimuli consisted of three Chinese words for different colors (“
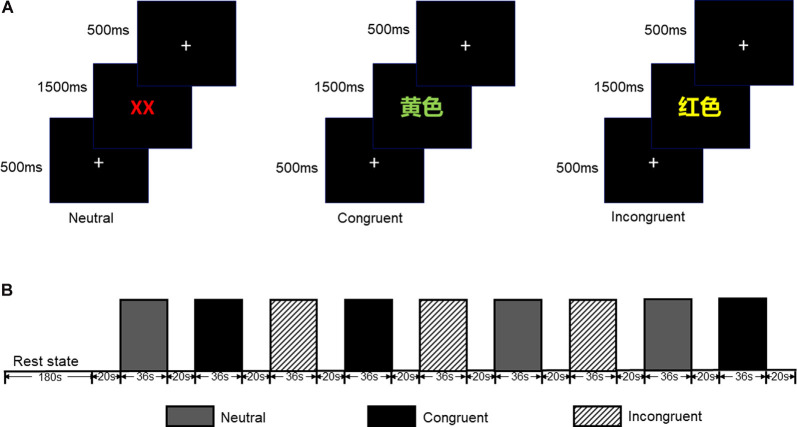
” for red, “
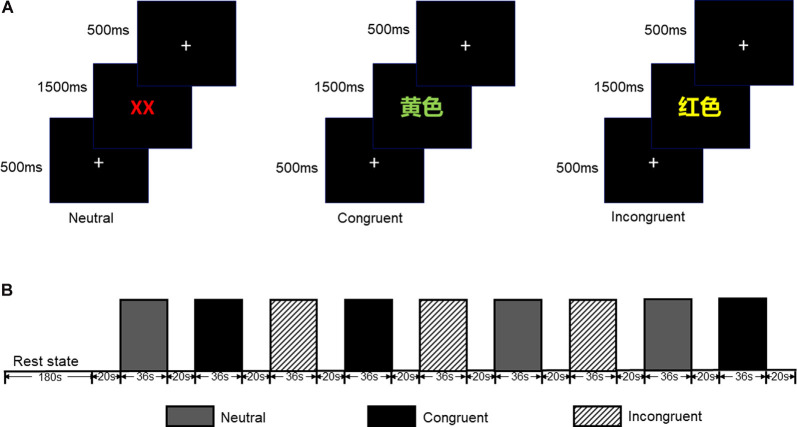
” for green, and “
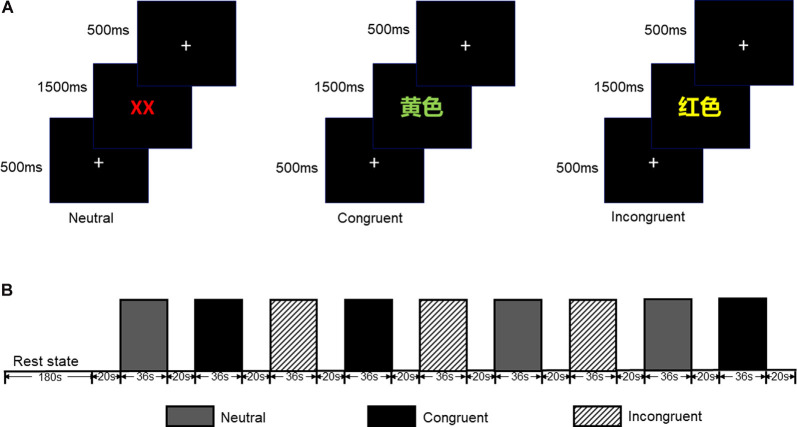
” for yellow,) and a neutral stimulus (an English letter “X”; Figure 1). Every condition contained three blocks, and a total of nine test blocks of three conditions were displayed in random order. There was a 2 s cue to alert participants before each rest block, and 20 s rest blocks separated the test blocks. For matching three colors (red, green, and yellow) randomly in equilibrium, 18 trials of each test block were sequentially displayed at random. Every trial lasted 1.5 s with 0.5 s intervals between each test stimulus. Participants were required to identify the font color quickly and to make sure of the accuracy of their reaction.

#### Shifting Attention Test (SAT)

**Figure 2:**
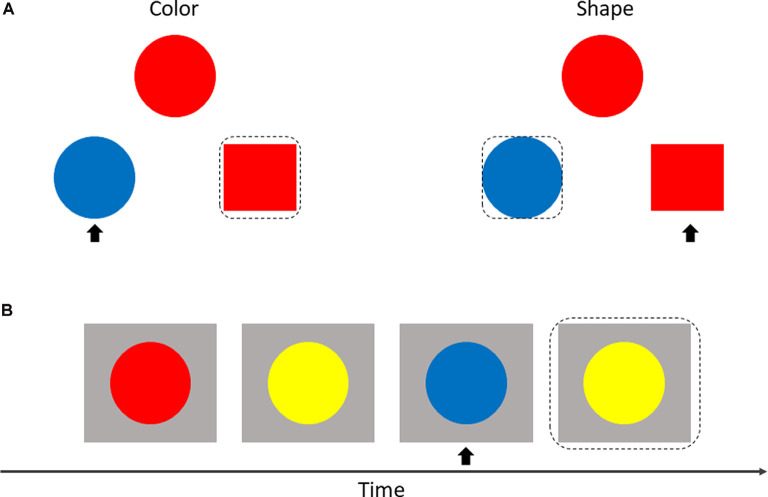
Display of shifting attention test (SAT; **A**) and 2-back test **(B)**. The matched object is within a black dotted box, and the non-matched object is marked with a black arrow.

The SAT measures cognitive flexibility by randomly switching between two instructions during a task. As shown in Figure 2A, three geometric objects were displayed on the screen at the same time with an instruction (“color” or “shape”). Participants were asked to match objects at the bottom of the screen (on the left or right) to the object at the top of the screen according to the property described in the instruction. Each of the three objects was given different properties at random: color (red or blue) × shape (square or circle). Participants responded by pressing the keys “←” or “→” which represented the left or right object at the bottom of the screen.

#### 2-Back Test

The adaptive color 2-back test was used to assess the effect of working memory. In this test, circles with different colors were presented sequentially at the center of the screen. Participants were invited to make a response by pressing a key (space bar) when the current color of the circle matched the color of the circle in two presentations previously (Figure 2B).

### HD-tDCS

**Figure 3:**
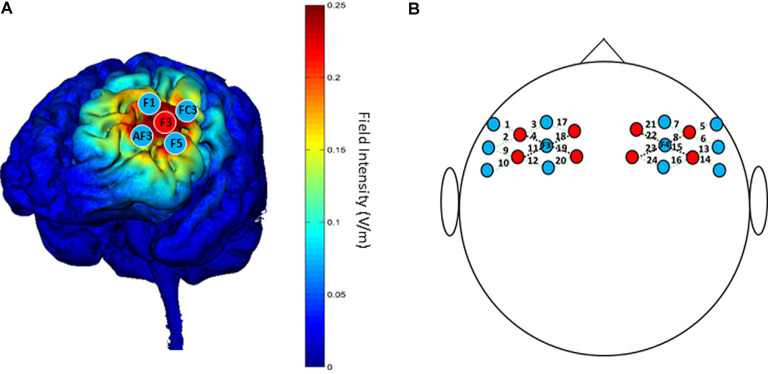
**(A)** “Red circles” indicate positions of anodal electrodes; “Blue circles” indicate positions of cathodal electrodes; the theoretical current intensity on the cortex (left DLPFC) of the tDCS electrode array is predicted by Soterix HD-Explore. **(B)** “Red circles” indicate positions of emitters; “Blue circles” indicate positions of detectors. The 24 channels are shown by dotted lines paired with numbers. The four channels surrounding F3/F4 that are represented by black dotted lines are used for analysis.

A battery-powered constant current DC stimulator (1300A&4×1-C3A, Soterix Medical, New York, NY, USA) was used to deliver 1.5 mA HD-tDCS stimulation in each session. According to the international 10-10 EEG System, the anodal electrode was placed on the scalp location F3, and four cathodal electrodes were placed over AF3, F1, F5, and FC3; theoretical current intensity at the cortex (left DLPFC) with this tDCS electrode array is shown in Figure 3A. The conductive gel was placed on the scalp under the hair to ensure connectivity before stimulating. The current intensity was set as 1.5 mA in deference to the limited tolerance of participants.

### fNIRS

The NTS fNIRS system used in this study (Gowerlabs, UK) employed continuous-wave near-infrared ranged from 780 to 850 nm. The unit contained eight laser-diode sources and 12 detectors that were tested with the UCL optical topography system (Everdell et al., 2005). The probe arrays were designed to cover the DLPFC and nearby brain regions, which allowed for 24 different channels with identical 3.0 cm separations between each couple source and detector, except long-distance separations between source and detector (Figure 3B). To ensure the accuracy of measurements, the position of probes was confirmed by the international 10-20 system, with two detectors placed at F3 and F4 separately.

### Design and Procedure

**Figure 4:**
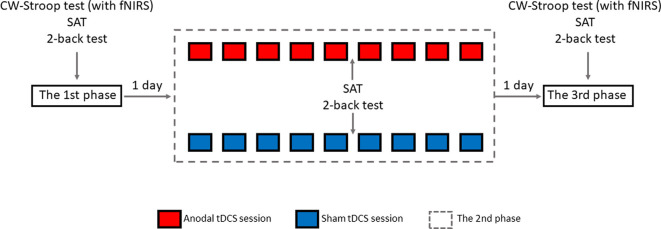
Overview of study design.

The experimental design was randomized, single-blinded, and sham-controlled. As shown in Figure 4, there were three phases in the experiment: pre-intervention (baseline), HD-tDCS sessions, and post-intervention. In the 1st phase, 1 day before the 2nd phase, all participants were invited to finish the CW-Stroop test (under fNIRS), SAT, and 2-back test. There were an experimental instruction and an initial practice session (1–2 min, 30–60 trials) before each test in this phase. In the 2nd phase, nine HD-tDCS sessions were undertaken every other day across 18 days, and participants were asked to complete the SAT and 2-back test on the day after the 5th HD-tDCS session. There was no CW-Stroop test (under fNIRS) after the 5th HD-tDCS session because the experimental schedule conflicted with the curriculum plans of the participants who had no enough time to complete the CW-Stroop test (under fNIRS). The 3rd phase was the day after the 2nd phase, and the content of the 3rd phase was identical to the 1st phase. For every HD-tDCS session, the anodal group was asked to accept anodal HD-tDCS stimulation on the left DLPFC for 20 min. The sham group was asked to accept sham HD-tDCS stimulation which was applied at 1.5 mA just for 1 min, including 30 s at the beginning for ramping up to 1.5 mA, and 30 s at the end for ramping down. It is worth mentioning that the participants were asked to declare whether they had a mood abnormality or intolerance of stimuli during the experiment. All participants finished the experiment and reported no adverse effects except a slight skin tingling. Additionally, participants reported whether they identified the sham or active HD-tDCS condition: all participants believed themselves to have undergone real stimulation.

### Data Processing

For behavioral performance, the Stroop effect was calculated by subtracting reaction time or error rate for the neutral condition from those values for the incongruent condition in the CW-Stroop test (Vitkovitch et al., 2002). The score for the SAT was calculated as accuracy (%)/reaction time (ms). Score for the 2-back test was calculated as:

(the number of correct responses−the number of wrong responses)/total number of responses

The raw data of fNIRS was filtered by band-pass filtering with cutoff frequencies of 0.01 and 0.1 Hz to eliminate physiological noise and baseline drift. Using the modified Beer–Lambert law (Sassaroli and Fantini, 2004; Kocsis et al., 2006; Baker et al., 2014), the concentration changes for HbO, HbR, and total hemoglobin (HbT; as calculated by HbO + HbR were determined. The hemodynamics of the last 5 s of the inter-stimuli rest period (18 s) were used as a baseline to normalize hemodynamic changes during the 36 s task block. Individual HbO data was calculated by averaging three blocks for each condition, and HbO data at the group level was obtained by averaging all individual data. Previous studies have suggested that HbO is a more sensitive reflection of cortical activation than either HbR or HbT, so only HbO data were analyzed in this study (Hoshi, 2010).

Separate 2 × 3 repeated-measures ANOVAs with the factors “group” (anodal, sham) and “time” (pre-tDCS, after 5th tDCS session, post-tDCS) were used to assess the effect of HD-tDCS in the SAT and 2-back test. A 2 (group: anodal, sham) × 2 (time: pre-tDCS, post-tDCS) repeated measures ANOVA was used to assess the fNIRS and CW-Stroop test data. For the ANOVAs, effect sizes were additionally measured by calculating the partial eta squared ($\eta_{\text{p}}^{2}$, Cohen, 1973), and the guideline proposed by Cohen was followed to interpret $\eta_{\text{p}}^{2}$ [i.e., 0.01 (small effect), 0.09 (medium effect), 0.25 (large effect)]. The statistical significance of the results in our study was defined as *p*-value < 0.05. All analyses were performed using SPSS software v25.0.

## Results

### Baseline

As shown in Table 2, the independent samples *t*-tests revealed that there were no significant differences in reaction time or error rate in the CW-Stroop test between the two groups, nor were any significant differences detected in HbO concentration. Additionally, scores on the SAT and 2-back tests in the anodal group were not significantly different compared to the sham group.

**Table 2:** Mean value ± standard deviation of baseline level on Stroop effect, shifting attention test (SAT) and 2-back test in the anodal and sham groups.

	Anodal group (*n* = 22)	Sham group (*n* = 21)	*t*	*p*-value
Stroop effect				
Reaction time (ms)	73.39 ± 59.28	63.34 ± 45.68	0.62	0.54
Error rate (%)	0.50 ± 2.64	0.65 ± 2.87	−0.18	0.86
HbO on lDLPFC (μmol)	0.05 ± 0.21	0.00 ± 0.23	0.64	0.53
HbO on rDLPFC (μmol)	0.03 ± 0.30	0.05 ± 0.20	−0.17	0.87
SAT (ms^−1^)	1.12e-3 ± 0.11e-3	1.14e-3 ± 0.12e-3	−0.68	0.50
2-back test (%)	27.11 ± 25.79	16.90 ± 16.15	1.55	0.13

### Stroop Effect

**Figure 5:**
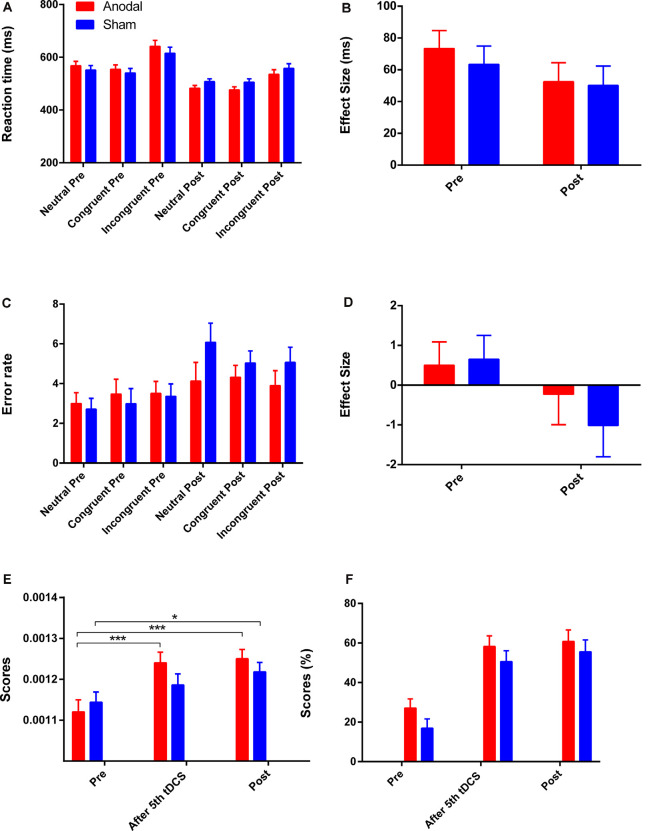
The scores on CW-Stroop test, SAT, and 2-back test. **(A,B)** Mean reaction times for each of the three conditions and the Stroop effect in the CW-Stroop test. **(C,D)** Mean error rates for each of the three conditions and the Stroop effect in the CW-Stroop test. **(E)** Variation in scores for the SAT at three time points. **(F)** Variation in scores for the 2-back test at three time points. “Pre” refers to pre-tDCS (the 1st phase), and “post” refers to post-tDCS (the 3rd phase). All values are presented as mean ± SEM. Bonferroni-adjusted contrast: *p < 0.05, ***p < 0.001.

The reaction time on all three conditions was improved in both groups (Figure 4A). A greater reduction in Stroop effect on reaction time was observed in the anodal group (mean change = 20.97, SEM = 13.56) compared to the sham group (mean change = 13.25, SEM = 13.88), but the effects of time (*F*_(1,41)_ = 3.11 *p* = 0.09, $\eta_{\text{p}}^{2}$ = 0.07), group (*F*_(1,41)_ = 0.21, *p* = 0.65, $\eta_{\text{p}}^{2}$ = 0.005), and interaction of time and group (*F*_(1,41)_ = 0.16, *p* = 0.69, $\eta_{\text{p}}^{2}$ = 0.004) were found to be not significant after a repeated measures ANOVA. An increase in error rate for each condition was observed in both groups (Figure 5C), and a repeated measures ANOVA found a greater reduction in Stroop effect for the sham group (mean change = 1.67, SEM = 0.83) compared the anodal group (mean change = 0.73, SEM = 0.81) with a significant main effect of time (*F*_(1,41)_ = 4.30, *p* = 0.04, $\eta_{\text{p}}^{2}$ = 0.10), but there were not significant effects of group, or interaction of time and group (all *p* > 0.05). Moreover, the separate Student’s *t*-tests revealed a significant increase in error rate for the neutral and congruent conditions in the sham group (*t*_neutral_ = −2.76, *p* = 0.01; *t*_congruent_ = −3.04, *p* = 0.01; *t*_incongruent_ = −2.06, *p* = 0.05) but there were no significant changes on all three conditions in the anodal group (*t*_neutral_ = −1.57, *p* = 0.13; *t*_congruent_ = −0.93, *p* = 0.37; *t*_incongruent_ = −0.50, *p* = 0.62). Therefore, the lowest Stroop effect on error rate was found in the sham group due to the excessive error rate (post-tDCS) for the neutral condition. Mean actual reaction times and error rates for the three conditions and the Stroop effect are presented in Figures 5A–D.

### SAT

For the SAT test, repeated measures ANOVA showed a significant effect of time (*F*_(2,82)_ = 25.66, *p* < 0.001, $\eta_{\text{p}}^{2}$ = 0.39), and a significant interaction effect between group and time (*F*_(2,82)_ = 3.60, *p* = 0.03, $\eta_{\text{p}}^{2}$ = 0.08). For the simple effect, Bonferroni-adjusted contrast showed that the scores of the last two time points (after 5th tDCS session, post-tDCS) were all significantly different compared to the baseline in the anodal tDCS group (all *p* < 0.001), and the significant improvement for the sham group was just observed at post-tDCS (Figure 5E).

### 2-Back Test

There were improvements in the 2-back score in both groups with a significant main effect of time (*F*_(2,82)_ = 52.31, *p* < 0.001, $\eta_{\text{p}}^{2}$ = 0.56) after repeated measures ANOVA, but the effects of group and interaction of time and group were not significant (all *p* > 0.05). For the main effect of time, the further Bonferroni-adjusted contrast showed significant enhancement on performance from baseline to the time point “after 5th tDCS session” and “post-tDCS” regardless of the difference of group. The variation of scores on the 2-back test is shown in Figure 5F.

### fNIRS

**Figure 6:**
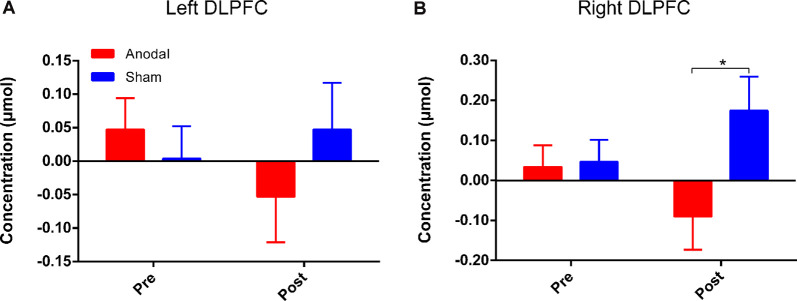
HbO concentrations for Stroop effect over left DLPFC **(A)** or right DLPFC **(B)**. “Pre” refers to pre-tDCS (the 1st phase), and “post” refers to post-tDCS (the 3rd phase). All values are presented as mean ± SEM. Bonferroni-adjusted contrast: *p < 0.05.

Four channels surrounding position F3/F4 were averaged as one channel to represent the DLPFC cortex (Figure 3B). In order to avoid interference from variations in local skin blood flow and futile hemodynamic changes during the CW-Stroop test, the correlation between the Stroop effect and HbO was calculated by subtracting the HbO concentration measured during the neutral condition block from that measured during the incongruent condition block. For the left DLPFC, although there were no significant effects of time (*F*_(1,41)_ = 0.28, *p* = 0.60, $\eta_{\text{p}}^{2}$ = 0.007), group (*F*_(1,41)_ = 0.19, *p* = 0.67, $\eta_{\text{p}}^{2}$ = 0.005), and interaction between group and time (*F*_(1,41)_ = 1.85, *p* = 0.18, $\eta_{\text{p}}^{2}$ = 0.04) after performing two factors (“group”: anodal, sham; “time”: pre tDCS, post tDCS) repeated measures ANOVA, a decrease in HbO was observed in the anodal group but not in the sham group (Figure 6A). For the right DLPFC, there was only a significant interaction effect between two factors (*F*_(1,41)_ = 4.41, *p* = 0.04, $\eta_{\text{p}}^{2}$ = 0.10) of the Stroop effect by repeated measures ANOVA, but the main effects of time (*F*_(1,41)_ = 0.001, *p* = 0.97, $\eta_{\text{p}}^{2}$ < 0.001) and group (*F*_(1,41)_ = 3.00, *p* = 0.09, $\eta_{\text{p}}^{2}$ = 0.07) were not significant. For the simple effect on the right DLPFC, Bonferroni-adjusted contrast showed a significantly lower HbO associated with the Stroop effect in the post-tDCS anodal group compared to that in the sham group (Figure 6B).

## Discussion

The present study examined the effects of repeated anodal HD-tDCS over the left DLPFC on executive functions. According to the theoretical model of executive function, its three major components (inhibitory control, working memory, and cognitive flexibility) are closely connected with the PFC (Funahashi, 2001; Diamond, 2012). Furthermore, anodal tDCS is known to improve executive performance through weak current stimulation of the left DLPFC (Strobach and Antonenko, 2017). To our knowledge, this study is the first tDCS research to examine the influence of multiple active HD-tDCS interventions on executive functions, and the main results of our study showed that 9 anodal HD-tDCS sessions can improve different aspects of executive functions.

Inhibitory control is the ability to focus on specific stimuli but suppress others, and is assumed to be closely interrelated with the processes of working memory and cognitive flexibility (Davidson et al., 2006; Blakey et al., 2016; Chmielewski et al., 2016). This study used the Stroop effect (incongruent-neutral) as a probe of inhibitory control (Vitkovitch et al., 2002). Although a decrease in the Stroop effect on reaction time was found, the changes were not statistically significant. A recent study similarly suggested that anodal tDCS does not provide benefits concerning Stroop interference (Baumert et al., 2020). Intriguingly, the Stroop effect on error rate decreased in both groups. However, further analysis showed that a significantly higher error rate for the neutral condition resulted in a reduction in the Stroop effect (incongruent-neutral) on the error rate in the sham group. In other words, the improvement in reaction time for each condition comes at the expense of an increase in error rate for the sham group. This means that the speed-accuracy tradeoff which was found to be optimized by exercises (Balci et al., 2011) may not be changed positively in a sustained fashion over time after sham tDCS interventions, but can be optimized in the anodal tDCS group. This is only a conjecture; these results may be due to the limitations of the Stroop test, and this issue should be addressed in further research.

Cognitive flexibility and working memory have been found to obtain benefits from active tDCS (Metuki et al., 2012; Nikolin et al., 2015). There have been few studies to examine how long-term repeated anodal HD-tDCS affects working memory and cognitive flexibility. Some evidence indicates that anodal tDCS over the left DLPFC can enhance cognitive flexibility (Borwick et al., 2020), and the results of our study supported this conclusion that multiple anodal HD-tDCS sessions can improve cognitive flexibility significantly in comparison to the sham group. Additionally, the previous study has examined that the effect of multiple tDCS sessions was continuous and robust (Im et al., 2019). A significant improvement in cognitive flexibility was shown in the anodal group after the 5th tDCS session, which was earlier than that in the sham group, and this might be related to cumulative effects of repeated modulation of HD-tDCS sessions. However, significant improvements in working memory were not observed in the anodal group compared with the sham group. These results are similar to those found previously (Nikolin et al., 2019), and there may be two potential reasons for this: (A) Working memory is thought to consist of a series of different sub-processes that involve more than one brain region, although the DLPFC is indispensable to working memory (Öztekin et al., 2009; Barbey et al., 2013). (B) There may be a ceiling effect on the 2-back test because this test might be relatively simple for participants who were undergraduates with high cognitive capability.

Long-term potentiation (LTP) is an enduring enhancement of synaptic connections, which underlies processes of behavioral performance such as learning and memory (Rioult-Pedotti et al., 2000). Long-lasting repeated tDCS is thought to induce late LTP-like plasticity of the functional cortex (Monte-Silva et al., 2012). Additionally, according to the theory of neurovascular coupling, changes in hemodynamics reflect the degree of activation of cortical regions (Pinti et al., 2020). We calculated Stroop effect-related hemodynamic changes by fNIRS and found lower HbO concentrations over the bilateral DLPFC after HD-tDCS intervention in the anodal group compared to that in the sham group. This may mean that unilateral intervention over one hemisphere could induce an efficient neuronal transmission between cerebral hemispheres. A previous study has also suggested that bilateral sensorimotor cortex (SMC) activation was reduced by anodal HD-tDCS over the left SMC (Muthalib et al., 2016). However, the results of the present study showed that the decrease in Stroop effect-related HbO concentrations was statistically significant over the right DLPFC, but not over the left DLPFC. Numerous studies have shown that the PFC is activated during tasks involving Stroop interference, and the activation over the left hemisphere is more prominent (Schroeter et al., 2004a,b). Lower activation over bilateral DLPFC is required during Stroop interference because the ability of inhibitory control is improved by multiple anodal HD-tDCS sessions, but the left DLPFC still must maintain adequate activation to ensure the functionality of inhibitory control. The close relations between left and right DLPFC should play a significant role in the Stroop-related process according to the rule of interhemispheric cooperation (Banich, 1998; Scalf et al., 2009), and the interhemispheric connectivity has proven to be enhanced by HD-tDCS (Yaqub et al., 2018). Therefore, we hypothesized that the right DLPFC, acting in an assistant role, is not necessarily required to be involved in the CW-Stroop test after HD-tDCS interventions, and thus the Stroop effect-related HbO concentration in the right DLPFC was reduced significantly compared to that in the sham group. However, this point should be examined by using tasks with different levels of difficulty in future experiments. The results of fNIRS might suggest that HbO concentration is a more sensitive measure than behavioral performance, and that hemodynamic response can be considered as an early predictive factor that reflects cortical activation modulated by repeated anodal HD-tDCS.

This study has some limitations that should be noted. The timing of the measurement of executive functions was meaningful for the present study. Previous studies have found that the effect of a single HD-tDCS session on cortical plasticity lasted at least 2 h (Kuo et al., 2013). To ensure that the benefit on cognitive function was induced by the accumulative effect of repeated HD-tDCS sessions, and not merely by the effect of the last single HD-tDCS session, all assessments of executive functions were carried out 1 day after stimulation, as in previous studies (Im et al., 2019; Molavi et al., 2020). However, the question of how to select a precise time point to assess the accumulative effect of multiple HD-tDCS sessions should be an interesting and worthwhile direction to explore further for future research. Although the DLPFC is crucial for executive functions, executive control has different degrees of connection with the entire PFC (Funahashi, 2001; Koechlin, 2003). Some information from other cortical areas might have been missed as only the DLPFC was examined in this study. The SAT and 2-back tests were carried out three times, while the CW-Stroop test with fNIRS was measured only twice, and this limitation was the result of a conflict between the experimental schedule and the curriculum plans of the participants who had no enough time to complete the CW-Stroop test with fNIRS. It would be worthwhile to employ other tasks with varying cognitive loads to assess performance in participants with different cognitive abilities. Also, a single-blind design in the present study would make the power of results weak. The gender difference in the experiment was not analyzed due to the limitation of sample size, which should be further examined in a future study with a sufficient sample size.

In summary, this study found that the left DLPFC is a cortical region that can be efficiently targeted for modulation by anodal HD-tDCS (nine tDCS sessions across 18 days) to significantly improve the cognitive flexibility of healthy participants, and provided effective intervention protocols which might be considered for use with patients with defective executive functions. Our results justify the claim that Stroop effect-related changes in hemodynamics in the DLPFC are a more sensitive way to assess inhibitory control than behavioral performance, and can be used as a potential effective indicator of clinical long-term tDCS treatment in future research with clinical samples. Further studies should expand on these results by stimulating different specific cortical regions related to executive functions or examining the effect of HD-tDCS employing various neuroimaging tools, such as electroencephalography (EEG) with high temporal resolution and fMRI with high spatial resolution.

## Data Availability Statement

The original contributions presented in the study are included in the article/Supplementary Material, further inquiries can be directed to the corresponding author/s.

## Ethics Statement

The studies involving human participants were reviewed and approved by Ethics Committee of Tangdu Hospital. The patients/participants provided their written informed consent to participate in this study.

## Author Contributions

HL, YG, PH, and YZ designed and conducted the study, including participant recruitment, data collection, and data analysis. HL and XY prepared the manuscript draft with important intellectual input from ZG. XZ provided editorial support during the preparation of this manuscript. All authors contributed to the article and approved the submitted version.

## Conflict of Interest

The authors declare that the research was conducted in the absence of any commercial or financial relationships that could be construed as a potential conflict of interest.
